# Bacterial Interference With Lactate Dehydrogenase Assay Leads to an Underestimation of Cytotoxicity

**DOI:** 10.3389/fcimb.2020.00494

**Published:** 2020-09-15

**Authors:** Sara Van den Bossche, Eva Vandeplassche, Lisa Ostyn, Tom Coenye, Aurélie Crabbé

**Affiliations:** Laboratory of Pharmaceutical Microbiology, Ghent University, Ghent, Belgium

**Keywords:** lactate dehydrogenase assay, cytotoxicity, host-pathogen interactions, respiratory infections, false negative (FN), *Klebsiella pneumoniae*, *Pseudomonas aeruginosa*

## Abstract

Models to study host-pathogen interactions *in vitro* are an important tool for investigating the infectious disease process and evaluating the efficacy of antimicrobial compounds. In these models, the viability of mammalian cells is often determined using the lactate dehydrogenase (LDH) cytotoxicity assay. In the present study we evaluated whether bacteria could interfere with the LDH assay. As a model for host-pathogen interactions, we co-cultured lung epithelial cells with eight bacteria encountered in the lower respiratory tract. We show that LDH activity is affected by *Pseudomonas aeruginosa, Klebsiella pneumoniae, Stenotrophomonas maltophilia*, and *Streptococcus pneumoniae*, and that this depends on the density of the start inoculum and the duration of infection. Two different mechanisms were discovered through which bacteria interfered with LDH activity, i.e., acidification of the cell culture medium (by *K. pneumoniae* and *S. pneumoniae*) and protease production (by *P. aeruginosa* and *S. maltophilia*). In addition, we developed and validated a modified protocol to evaluate cytotoxicity using the LDH assay, where bacterial interference with LDH quantification is avoided.

## Introduction

Lower respiratory tract infections are part of the top 10 global causes of death, leading to 3 million deaths per year worldwide (Sheffield, 2017). In the battle against infectious diseases, models that allow studying interactions between host and pathogen are important tools to examine the course of disease and to explore new therapies. An important parameter in these *in vitro* models is the evaluation of bacterial and/or antimicrobial agent cytotoxicity (Barnes et al., 2015).

Different assays based on permeability of the cell membrane or on cellular metabolic activity are available to determine cell viability. The latter tests, including the MTT assay [3-(4,5-dimethylthiazol-2-yl)-2–5-diphenyltetrazolium bromide] (Mosmann, 1983) or the recently developed bioluminescent real-time viability assay (Duellman et al., 2015), enable to quantify enzymatic activity and thus cellular metabolism. Metabolic assays are used both for assessing viability of host cells and bacteria and are thus not suitable to assess host cell viability during host-pathogen interactions (Grela et al., 2018). Permeability assays on the other hand, are based on the loss of cell membrane integrity in apoptotic and/or necrotic cells. These assays analyze the in- or exclusion of fluorescent or light-absorbing molecules (e.g., trypan blue exclusion assay, propidium iodide assay) or the release of intracellular enzymes upon cell lysis (e.g., lactate dehydrogenase assay) (da Costa et al., 1999). The lactate dehydrogenase (LDH) assay is based on the release of the intracellular enzyme LDH upon cell lysis, which is associated with apoptosis or necrosis. Furthermore, LDH is considered a stable enzyme. Indeed, high stability (i.e., retention of more than 70% of total activity) of LDH has been described after 45 days of storage at 25°C and −20°C of human serum (Jacobs et al., 1986). Consequently, LDH activity in the supernatant of cells exposed to infectious agents and/or a treatment can be used as a direct measurement of cytotoxicity (Korzeniewski and Callewaert, 1983) and this assay has been extensively used in host-pathogen interactions studies (Slevogt et al., 2007; Pompilio et al., 2010; Mairpady Shambat et al., 2015; Zahlten et al., 2015; Crabbé et al., 2017). Major advantages of this assay are its reliability, time-efficiency and easy evaluation (Mukhopadhyay et al., 2004).

However, it needs to be emphasized that this approach only yields useful results if the amount of LDH released from cells and/or its activity are not influenced by the specific environmental conditions of the experiment. In the present study we investigated whether bacteria that are often recovered from the lower respiratory tract of patients with chronic lung disease (i.e., *Pseudomonas aeruginosa, Staphylococcus aureus, Klebsiella pneumoniae, Stenotrophomonas maltophilia, Streptococcus pneumoniae, Achromobacter xylosoxidans, Moraxella catarrhalis*, and *Rothia mucilaginosa*) could influence results obtained with the LDH assay when evaluating bacterial cell-based cytotoxicity in the context of host-pathogen interactions. Based on available literature, high cytotoxicity is generally expected after infection with the pathogens *P. aeruginosa, S. aureus, K. pneumoniae*, and *S. maltophilia* (Hawdon et al., 2010; Karaba et al., 2013; Wang et al., 2013; Wen et al., 2018). For *P. aeruginosa* and *S. aureus*, cytotoxicity toward airway epithelial cells was found to be strain-dependent (Hawdon et al., 2010; Strobel et al., 2016). Despite the pathogenicity of *S. pneumoniae, A. xylosoxidans*, and *M. catarrhalis*, low cytotoxicity toward airway epithelial cells has been reported (Slevogt et al., 2007; Mantovani et al., 2012; Weight et al., 2019), while to our knowledge no information is available on the cytotoxicity of the commensal *R. mucilaginosa*.

## Materials and Methods

### Bacterial Strains and Culturing Conditions

Eight bacterial strains, frequently isolated from the airways of patients suffering from lower respiratory tract infections, were included in the present study: *K. pneumoniae* (ATCC 13883), *S. maltophilia* (ATCC 13637), *P. aeruginosa* (ATCC 15692), *S. pneumoniae* (ATCC 33400), *S. aureus* (ATCC 25923), *R. mucilaginosa* (DSM 20746), *A. xylosoxidans* (ATCC 27061), and *M. catarrhalis* (DSM 11994). The clinical isolates of *P. aeruginosa*, E33, E90, and E94, are part of a collection obtained through the Early *Pseudomonas* Infection Control Observational (EPIC Obs) Study (Feltner et al., 2016). *K. pneumoniae, S. maltophilia, P. aeruginosa, S. aureus*, and *A. xylosoxidans* were cultured 16 h (250 rpm, 37°C) in Luria Broth (LB) for all experiments. *S. pneumoniae, M. catarrhalis*, and *R. mucilaginosa* were grown for 24 h (250 rpm, 37°C) in brain heart infusion (BHI) broth.

For bacterial growth curves, the total bacterial load at different time points was determined by quantifying the CFU/mL of both the planktonic fraction and the fraction attached to the plastic surface of the 96-well plate (i.e., biofilms). Quantification of the planktonic fraction was done by plating the supernatant. The attached bacterial cells were detached from the surface by two subsequent cycles of vortexing (900 rpm, 5 min) and sonication (5 min; Branson Ultrasonic bath). Afterwards, quantification was done by plating (Vandeplassche et al., 2017).

### Epithelial Cells and Culturing Conditions

The adenocarcinomic alveolar cell line A549 (ATCC CCL-185) or the immortalized bronchial epithelial cell line BEAS-2B (ATCC CRL-9609) were used for all infections. Monolayers of A549 cells were grown in T75 flasks for 5 days in GTSF-2 medium (HyClone, Logan, UT) supplemented with 1.5 g/L sodium bicarbonate (Sigma-Aldrich), 10% fetal bovine serum (FBS, Life Technologies), 2.5 mg/L insulin transferrin sodium selenite (Lonza) and 1% penicillin-streptomycin (Sigma-Aldrich) at 37°C under 5% CO_2_, until 80% confluency. Monolayers of BEAS-2B cells were grown in T75 flasks for 5 days in Roswell Park Memorial Institute (RPMI) medium (Life Technologies) supplemented with 10% FBS and 1% penicillin-streptomycin at 37°C under 5% CO_2_, until 80% confluency. Then, cells were detached with a 0.25% trypsin-EDTA solution (Life Technologies) for A549 cells and a 0.05% trypsin-EDTA solution (Life Technologies) for BEAS-2B cells, transferred into a 24-well plate and grown in antibiotic free GTSF-2 medium or RPMI medium until confluency was reached, corresponding with a final cell density of ~2.5 × 10 ^5^ cells/well (doubling time: 10 h). For the infection study, GTSF-2 medium without FBS and antibiotics was used for both cell lines.

### Infection Assay

Prior to infection, all bacterial cultures were centrifuged and resuspended in serum- and antibiotic free GTSF-2 medium (epithelial cell culture medium). Infection studies were performed at a targeted multiplicity of infection (MOI) of 10:1. Plates were incubated for 24 h at 37°C and 5% CO_2_.

### Cytotoxicity Assay Using the Conventional LDH Protocol and Fluorescence Microscopy

After infection, cell supernatant was collected and centrifuged (3,700 rpm, 15 min). LDH activity of the resulting supernatant, a direct measurement of the dead fraction of epithelial cells, was determined using the LDH detection kit (Sigma-Aldrich) following the manufacturer's instructions. Cytotoxicity was expressed as a percentage of an uninfected control treated with 1% triton X-100 (Sigma-Aldrich). Efficient lysis of cells was achieved through vigorous pipetting.

After removing the supernatant, cell monolayers were rinsed once with Hank's Balanced Salt Solution (HBSS, Life Technologies). For fluorescence microscopy, monolayer cells were fixed in 4% paraformaldehyde (Electron Microscopy Sciences) for 20 min and stained with 4′,6-Diamidino-2-Phenylindole (DAPI, Life Technologies) to visualize epithelial cell nuclei. Afterwards, three representative images were taken per well using an EVOS FL Auto Imaging System (Life Technologies) equipped with a 20x objective (final magnification: 368x). The number of adhering cells in each image was determined by enumerating epithelial cell nuclei using the image processing application ImageJ (LOCI, University of Wisconsin). For calculation of cell adherence, the average cell number of three images was calculated and compared to the uninfected control. Cell detachment was then calculated as the complement of the adherent fraction.

### Determining the Influence of Bacterial Species on LDH Activity

A defined concentration of rabbit muscle LDH (Sigma-Aldrich), equal to that released by 2.5 × 10 ^5^ A549 cells (i.e., 707 mU/mL), was added per well of a 96-well plate. A total volume of 250 μL of bacterial suspension in serum- and antibiotic free GTSF-2 medium was added per well at a density that equals an MOI of 10:1 (2.5 × 10 ^6^ bacterial cells/well) based on 2.5 × 10 ^5^ epithelial cells/well, unless stated otherwise. As these experiments do not include epithelial cells, the term “density of the start inoculum” is used instead of MOI. The plate was incubated for 24 h, unless other time frames are mentioned (37°C, 5% CO_2_). Afterwards, the content of each well was centrifuged (3,700 rpm, 15 min) and 5 μL of the resulting supernatant was used to determine the remaining LDH activity. LDH activity was measured using the LDH detection kit (Sigma-Aldrich) following the manufacturer's instructions. LDH activity was expressed as a percentage compared to a control without bacteria unless stated otherwise. When indicated, a bacterial protease inhibitor cocktail (Sigma-Aldrich) was added at the start of experiment. The final concentration of the protease inhibitors was: 920 nM 4-(2-aminoethyl)benzenesulfonyl fluoride hydrochloride (AESBF), 4,000 nM ethylenediaminetetraacetic acid (EDTA), 80 nM bestatin, 12 nM pepstatin A and 12 nM E-64. As the bacterial protease inhibitor cocktail was dissolved in DMSO (Fisher Scientific), a solvent control (0.004 % DMSO) was included.

### Determining the Influence of pH on LDH Activity

A defined amount of rabbit muscle LDH (Sigma-Aldrich), equal to the amount released by 2.5 × 10 ^5^ A549 cells (i.e., 707 mU/mL), was added to 1 mL of serum- and antibiotic free GTSF-2 medium at a pH range of 5.6–7.0. The pH was measured using a pH checker (Hanna instruments) and was adjusted using a 1 M HCl stock solution (Honeywell). After 30 min incubation at this pH, LDH activity was determined and compared to the activity in the original GTSF-2 medium (pH = 7.4–7.6).

### Cytotoxicity Assay Using the Modified LDH Protocol

After infection, cell monolayers were rinsed with HBSS (Life Technologies) and exposed to 1% triton X-100 (Sigma-Aldrich) combined with vigorous pipetting to obtain cell lysis. Subsequently, LDH activity was determined following the protocol described above. The viable fraction of infected cultures was expressed as a percentage from an uninfected control, also treated with a 1% triton X-100 solution and subjected to vigorous pipetting. Cell cytotoxicity was then calculated as the complement of this viable fraction.

The correlation between the number of attached cells and LDH release was determined by seeding A549 cells at different cell densities (range: 0.03125 × 10 ^5^ cells/well −0.50 × 10 ^5^ cells/well) in 24-well plates in antibiotic free GTSF-2 medium followed by incubation for 4 days which resulted in a final cell density range of 0.08 × 10 ^5^ cells/well to 7.6 × 10 ^5^ cells/well (37°C, 5% CO_2_, doubling time: 10 h). Afterwards cells were detached with a 0.25% trypsin-EDTA solution (Life Technologies). The total cell number was determined by a trypan blue exclusion assay followed by light microscopy, using 0.4% trypan blue solution (Sigma-Aldrich), according to the manufacturer's instructions. Cell suspensions were centrifuged (1,200 rpm, 6 min) and resuspended in a 1% triton X-100 solution. Efficient lysis of cells was achieved through vigorous pipetting. The LDH activity of the resulting supernatant was determined as described above.

The LLOD was determined using A549 cell suspensions, due to the limitations in growing monolayers at extremely low cell numbers. Starting from a confluent monolayer grown in a T75 flask, cells were detached with a 0.25% trypsin-EDTA solution. The total cell number was determined using a trypan blue exclusion assay, as mentioned above. Cells were centrifuged (1,200 rpm, 6 min) and resuspended at a concentration of 1 × 10^7^ cells/mL. A 10-fold dilution series of this cell suspension was made until 10 cells/mL. The resulting dilution series was centrifuged (1,200 rpm, 6 min) and resuspended in a 1% triton X-100 solution. Efficient lysis of cells was achieved through vigorous pipetting. The LDH activity of the resulting supernatant was determined as described above.

### Statistical Analysis

Statistical analysis was done using SPSS Statistics 24. All experiments were performed at least in biological triplicate. Normal distribution was verified by the Shapiro-Wilk test. Normally distributed data were assessed by an independent sample *t*-test, after verifying equality of variances by Levene's test, by a one-sample *t*-test or by a one-way ANOVA. Non-normally distributed data were analyzed using a Wilcoxon signed-rank test or by a Kruskall-Wallis one-way ANOVA. Linear correlation data were calculated by performing simple linear regression when assumptions of homoscedasticity and normal distribution of errors, were met.

## Results

### Conventional LDH Assay and Microscopic Analysis Yield Discrepant Results After Infection of A549 Cells With *K. pneumoniae, S. maltophilia*, and *P. aeruginosa*

**Figure 1:**
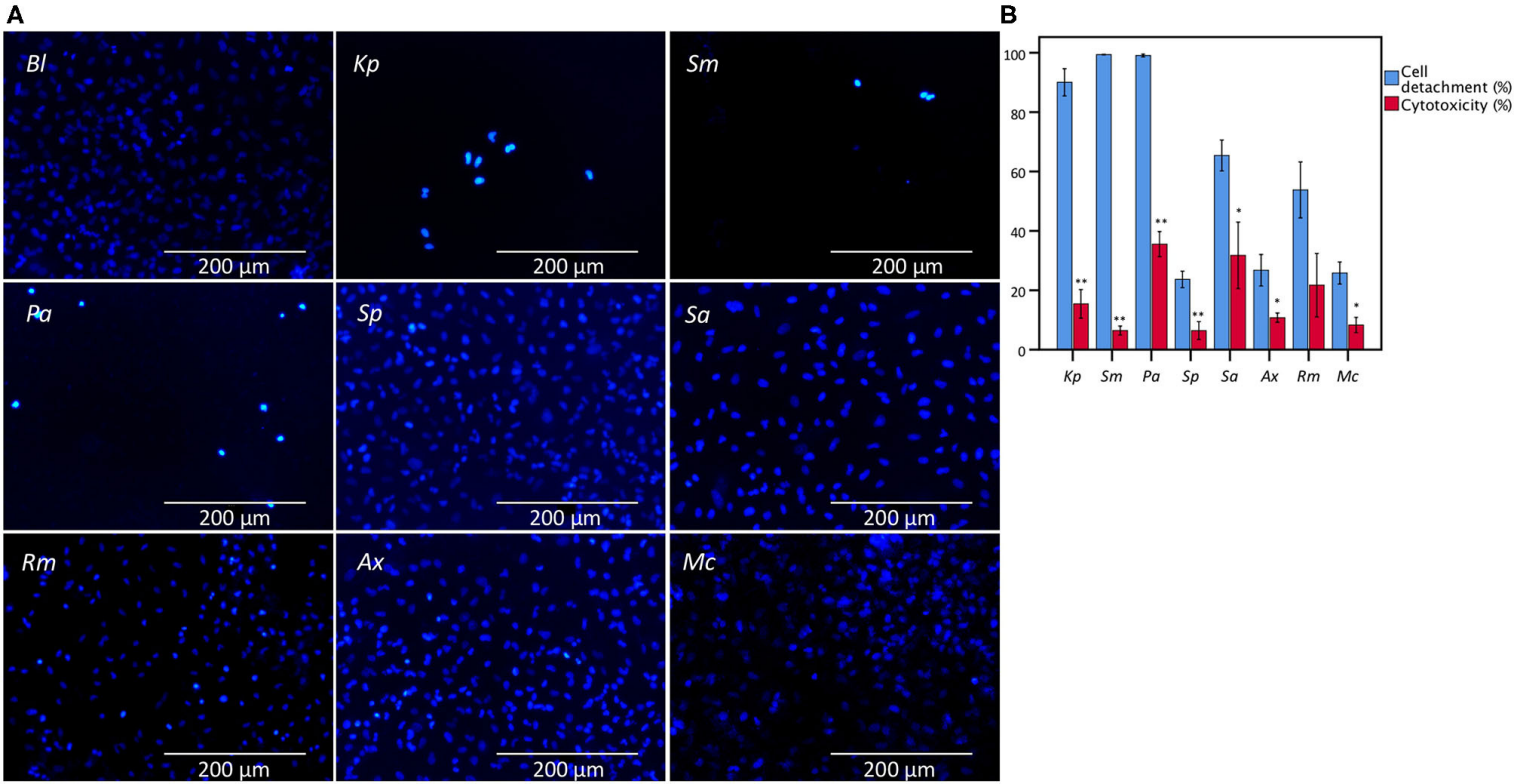
Comparison between the results of LDH cytotoxicity assay and cell detachment assessed by fluorescence microscopy following nuclei staining with DAPI of A549 cells infected with different bacteria from the lower respiratory tract. **(A)** Microscopic image (magnification: 368x) of an uninfected A549 monolayer (*Bl*) and after 24 h of infection with *K. pneumoniae* (*Kp*), *S. maltophilia* (*Sm*), *P. aeruginosa* (*Pa*), *S. pneumoniae* (*Sp*), *S. aureus* (*Sa*), *R. mucilaginosa* (*Rm*), *A. xylosoxidans* (*Ax*), and *M. catarrhalis* (*Mc*). **(B)** Comparison for A549 cells between cell detachment as determined by epithelial cell nuclei enumeration and cytotoxicity as determined by the conventional LDH assay. Cytotoxicity is expressed as percentage of signal obtained with an uninfected control treated with 1% triton X-100. Uninfected control (*Bl*), infected for 24 h with *Kp, Sm, Pa, Sp, Sa, Rm, Ax*, and *Mc* (*n* = 5–7, **p < 0.01; as determined by an independent sample *t*-test or a Mann-Whitney U test depending on the normality of the data). Error bars represent SE. *p < 0.05.

Bacterial cell-mediated cytotoxicity of A549 cells after 24 h of infection was determined for eight bacterial species encountered in patients suffering from lower respiratory tract infections (*K. pneumoniae, S. maltophilia, P. aeruginosa, S. pneumoniae, S. aureus, R. mucilaginosa, A. xylosoxidans*, and *M. catarrhalis*) using the conventional LDH assay, and compared with microscopic analysis of monolayer integrity by evaluating cell detachment. Significant differences were observed between cell detachment and LDH-based cytotoxicity values after infection with *K. pneumoniae, S. maltophilia, P. aeruginosa, S. pneumoniae, S. aureus, A. xylosoxidans*, and *M. catarrhalis* (Figure 1).

### *K. pneumoniae, S. maltophilia, P. aeruginosa, and S. pneumoniae* Affect LDH Activity

**Figure 2:**
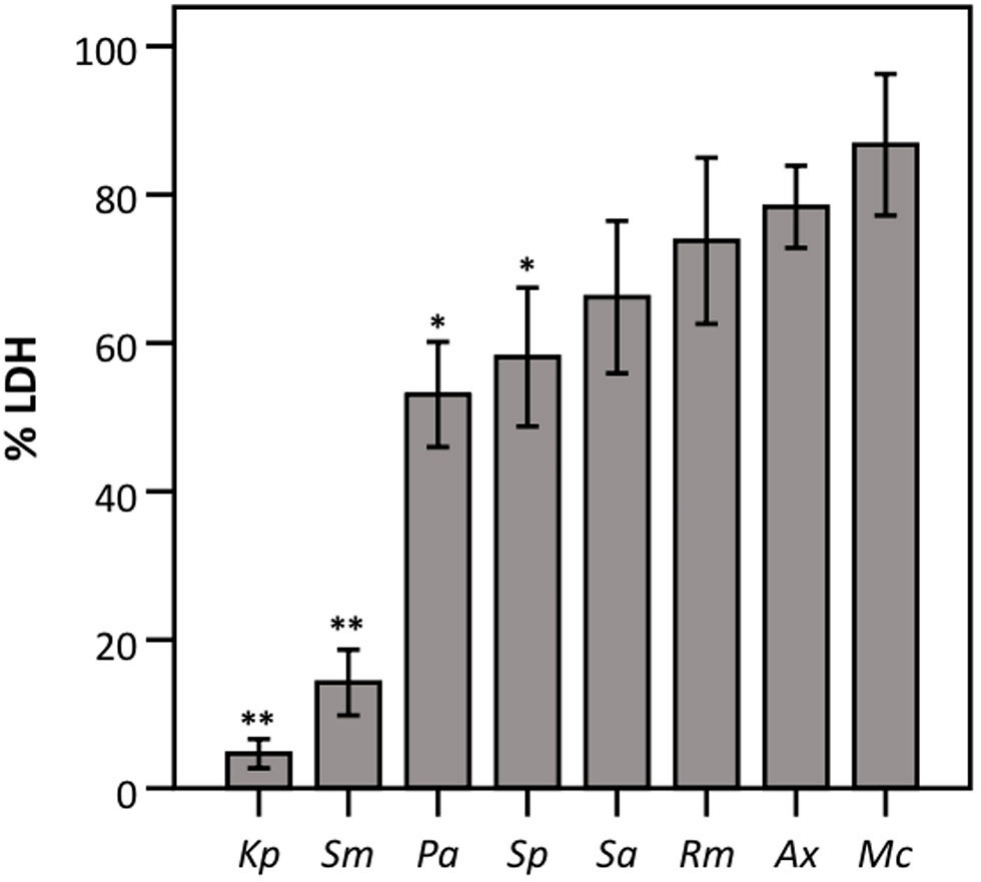
LDH activity after 24 h incubation with different bacteria (density of the start inoculum: 2.5 × 10 ^6^ CFU/well) expressed as a percentage compared to a control without bacteria. Results are shown for *K. pneumoniae* (*Kp*), *S. maltophilia* (*Sm*), *P. aeruginosa* (*Pa*), *S. pneumoniae* (*Sp*), *S. aureus* (*Sa*), *R. mucilaginosa* (*Rm*), *A. xylosoxidans* (*Ax*), and *M. catarrhalis* (*Mc*). Error bars represent SE. Significant differences with 100% are indicated (*n* = 6–7, *p < 0.05, **p < 0.01; as determined by a one sample *t*-test or a Wilcoxon signed-rank test depending on the normality of the data).

Subsequently, the influence of all eight bacterial species on LDH activity was assessed using a defined amount of commercially available LDH. As shown in Figure 2, the presence of *K. pneumoniae, S. maltophilia, P. aeruginosa*, and *S. pneumoniae* significantly reduced LDH activity, while no influence on LDH activity was observed for the other strains tested.

**Figure 3:**
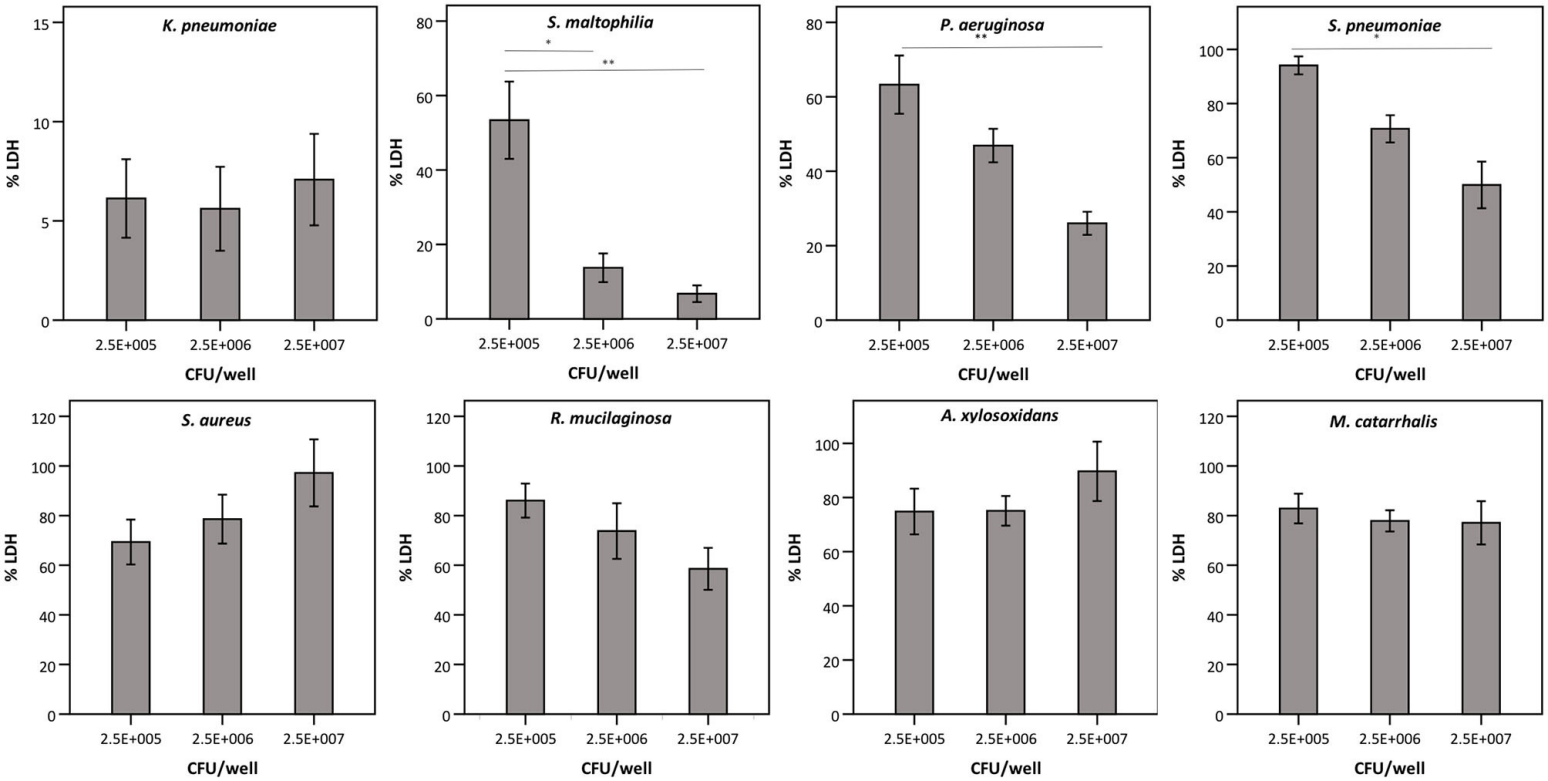
LDH activity after 24 h incubation with different bacteria (density of the start inoculum: 2.5 × 10^5^ CFU/well, 2.5 × 10^6^ CFU/well or 2.5 × 10^7^ CFU/well), expressed as a percentage compared to a control without bacteria. Results are presented for *K. pneumoniae, S. maltophilia, P. aeruginosa, S. pneumoniae, S. aureus, R. mucilaginosa, A. xylosoxidans*, and *M. catarrhalis*. Error bars represent SE. Significant differences between different start inocula are indicated (*n* = 6–7, *p < 0.05, **p < 0.01; as determined by one-way ANOVA followed by Bonferroni *post-hoc* analysis or by Kruskall-Wallis one-way ANOVA depending on the normality of the data).

Next, we evaluated whether the observed effect on LDH activity depends on the density of the start inoculum, incubation time, and/or the strain used. The term “density of the start inoculum” is used instead of MOI, as no airway epithelial cells were included in these experiments. Firstly, an inverse correlation was found between the density of the start inoculum and LDH activity for *S. maltophilia, P. aeruginosa*, and *S. pneumoniae* (Figure 3). In contrast, for *K. pneumoniae* all start inoculum densities tested resulted in the same effect on LDH activity. For *S. aureus, R. mucilaginosa, A. xylosoxidans*, and *M. catarrhalis* there was no influence on LDH activity after 24 h for the inoculum range tested, indicating that these species do not affect LDH activity at the tested start inocula (Figure 3).

**Figure 4:**
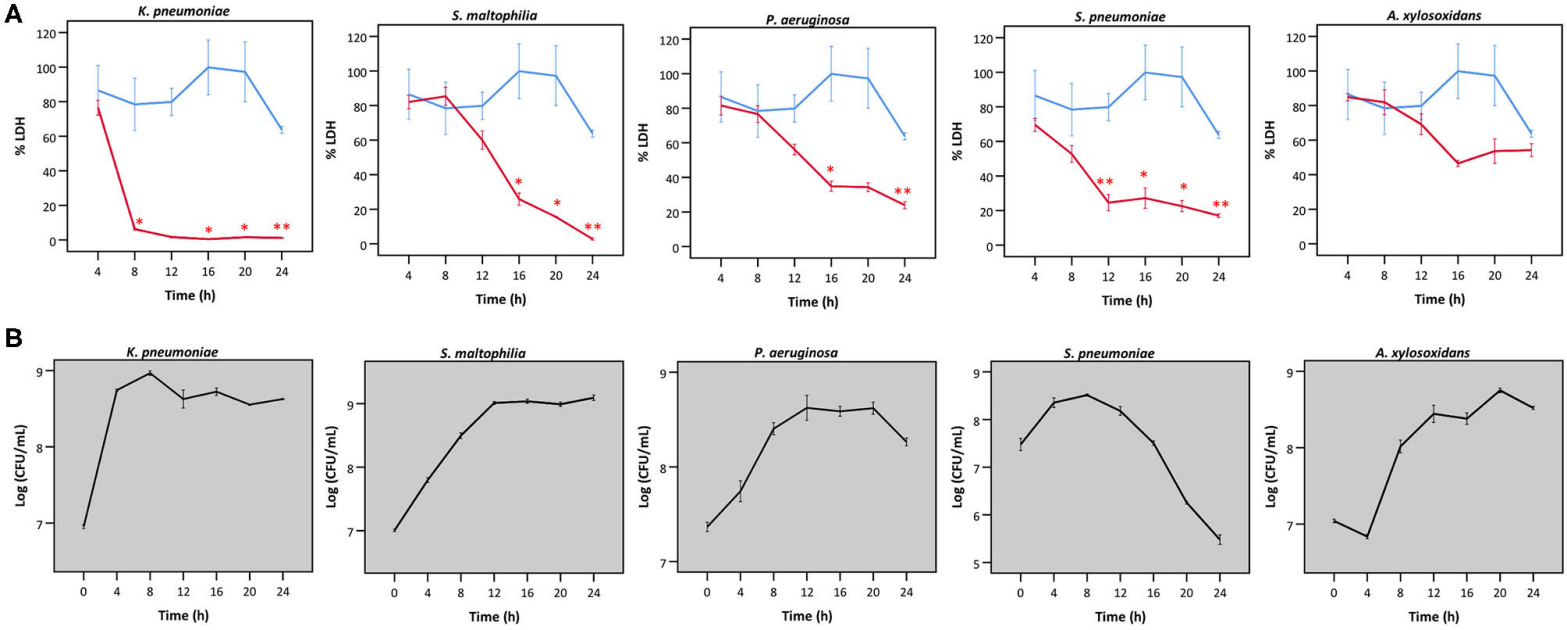
**(A)** LDH activity after incubation with different bacteria for different times, expressed as a percentage compared to LDH activity at start. Results are presented for *K. pneumoniae, S. maltophilia, P. aeruginosa, S. pneumoniae* and *A. xylosoxidans* in red; and for a control without bacteria in blue. Error bars represent SE. Significant differences with a control without bacteria are indicated (*n* = 3, *p < 0.05, **p < 0.01; as determined by an independent sample *t*-test or a Mann-Whitney U-test depending on the normality of the data). **(B)** Growth curves based on CFU/mL for *K. pneumoniae S. maltophilia, P. aeruginosa, S. pneumonia*, and *A. xylosoxidans*. Error bars represent SE.

Secondly, we queried whether the extent of bacterial interference with LDH activity depended on the incubation time (Figure 4A), hereby focusing on the species that interfered with LDH activity after 24 h (Figures 2, 3). *A. xylosoxidans* was included as a control as it showed no interference with LDH activity (Figures 2, 3). Simultaneously, the total bacterial load was determined over time, to evaluate if effects on LDH activity were linked to growth profiles (Figure 4B). For *P. aeruginosa* and *S. maltophilia*, a significant decrease in LDH activity was observed after 12–16 h, compared to a control without bacteria. For *K. pneumoniae* and *S. pneumoniae* a significant influence on LDH activity was detected after 8 and 12 h, respectively. For *S. pneumoniae*, we observed a decrease in bacterial load after 12 h incubation (Figure 4B), indicating that bacteria entered the death phase of growth. Nevertheless, interference of *S. pneumoniae* with LDH activity already started prior to the initiation of the death phase (Figures 4A,B). For comparison, the results for *A. xylosoxidans* are also shown. Although the growth of *A. xylosoxidans* was similar to the growth of bacterial species interfering with LDH activity, no significant differences in LDH activity were observed (Figures 4A,B).

**Figure 5:**
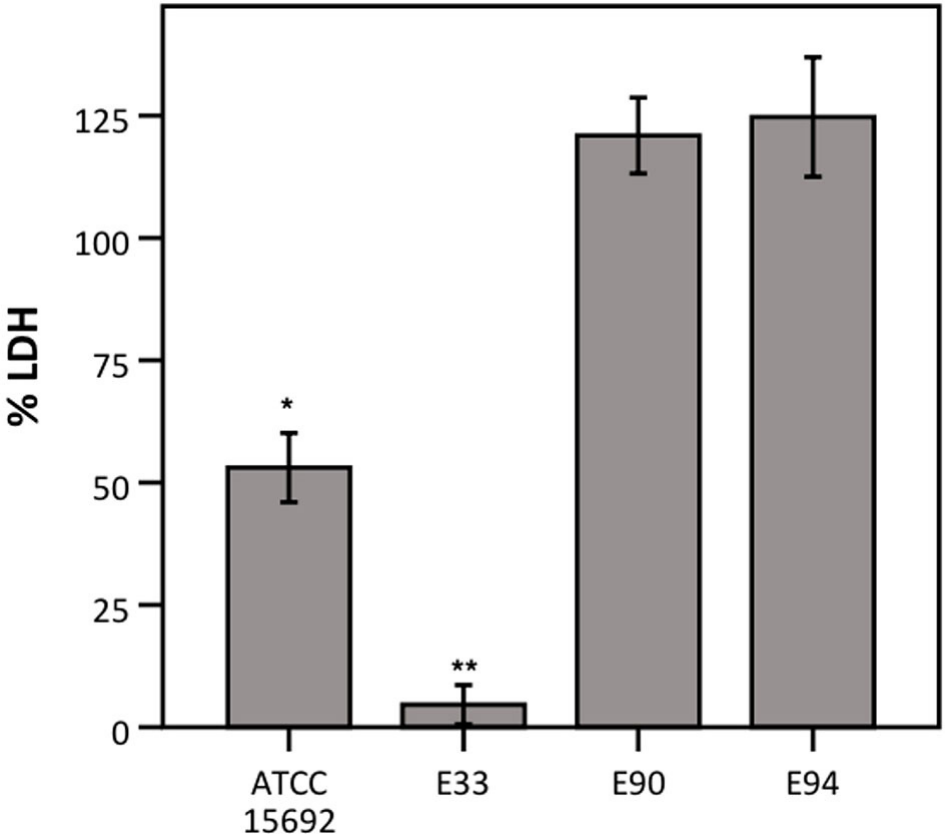
LDH activity after incubation with different strains of *P. aeruginosa*, expressed as a percentage compared to a control without bacteria. Results are presented for *P. aeruginosa* ATCC 15692, E33, E90, and E94. Error bars represent SE. Significant differences with 100% are indicated (*n* = 3–4, *p < 0.05, **p < 0.01; as determined by a one sample *t*-test or a Wilcoxon signed-rank test depending on the normality of the data).

Thirdly, different strains of *P. aeruginosa* were evaluated for their ability to influence LDH activity, as *P. aeruginosa*-mediated cytotoxicity has been reported to be strain-dependent (Hawdon et al., 2010). Therefore, LDH activity was determined after exposure to different strains of *P. aeruginosa*, i.e., three clinical cystic fibrosis isolates (E33, E90, and E94; all deficient in the quorum sensing regulator LasR which is a hallmark of late CF clinical isolates) and the reference strain (ATCC 15692; used in previous experiments and characterized by a functioning quorum sensing system). LDH activity was extensively affected by E33, moderately affected by the reference strain and not affected by E90 and E94 (Figure 5).

Taken together, interference with LDH activity by *K. pneumoniae, S. maltophilia, P. aeruginosa*, and *S. pneumoniae* likely explains the observed discrepant results between the conventional LDH assay and cell detachment (Figure 1). Finally, we evaluated if this discrepancy could also be observed using another lung epithelial cell line (BEAS-2B). Significant differences in this cell line were also observed after infection with *S. maltophilia* and *S. pneumoniae* (Figure S1).

### LDH Activity Is Affected by Acidification of the Cell Culture Medium and by Bacterial Proteases

**Figure 6:**
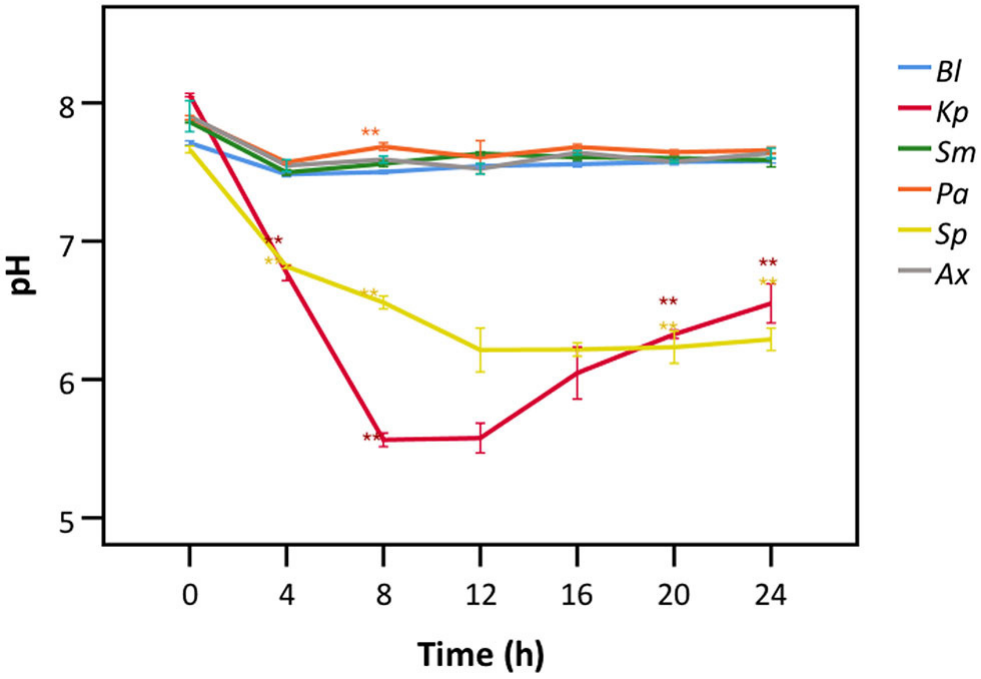
pH of cell culture medium after exposure to different bacteria for different time frames. Results are presented for a control without bacteria (*Bl*), *K. pneumoniae* (*Kp*), *S. maltophilia* (*Sm*), *P. aeruginosa* (*Pa*), *S. pneumoniae* (*Sp*), and *A. xylosoxidans* (*Ax*). Error bars represent SE. Significant differences with the control without bacteria are highlighted (*n* = 3, *p < 0.05, **p < 0.01; as determined by one-way ANOVA followed by Dunnett *post-hoc* analysis or by Kruskall-Wallis One-Way ANOVA depending on the normality of the data).

Hereafter, we aimed to understand the mechanism behind bacterial interference with LDH activity. Only the species showing interference with LDH activity after 24 h (Figures 2, 3) were included. *A. xylosoxidans* was added as a control as it did not interfere with LDH activity. A significant decrease in the pH of the cell culture medium was observed in the presence of *K. pneumoniae*, with the lowest value (pH = 5.6) being observed after 8 h (Figure 6). Correspondingly, LDH activity was strongly reduced after 8 h of bacterial presence (Figure 4A). The decline in pH was less pronounced after exposure to *S. pneumoniae* and resulted in a minimum pH value of 6.2 after 12 h (Figure 6). This was again in accordance with the major drop in LDH activity observed after 12 h for *S. pneumoniae* (Figure 4A).

**Figure 7:**
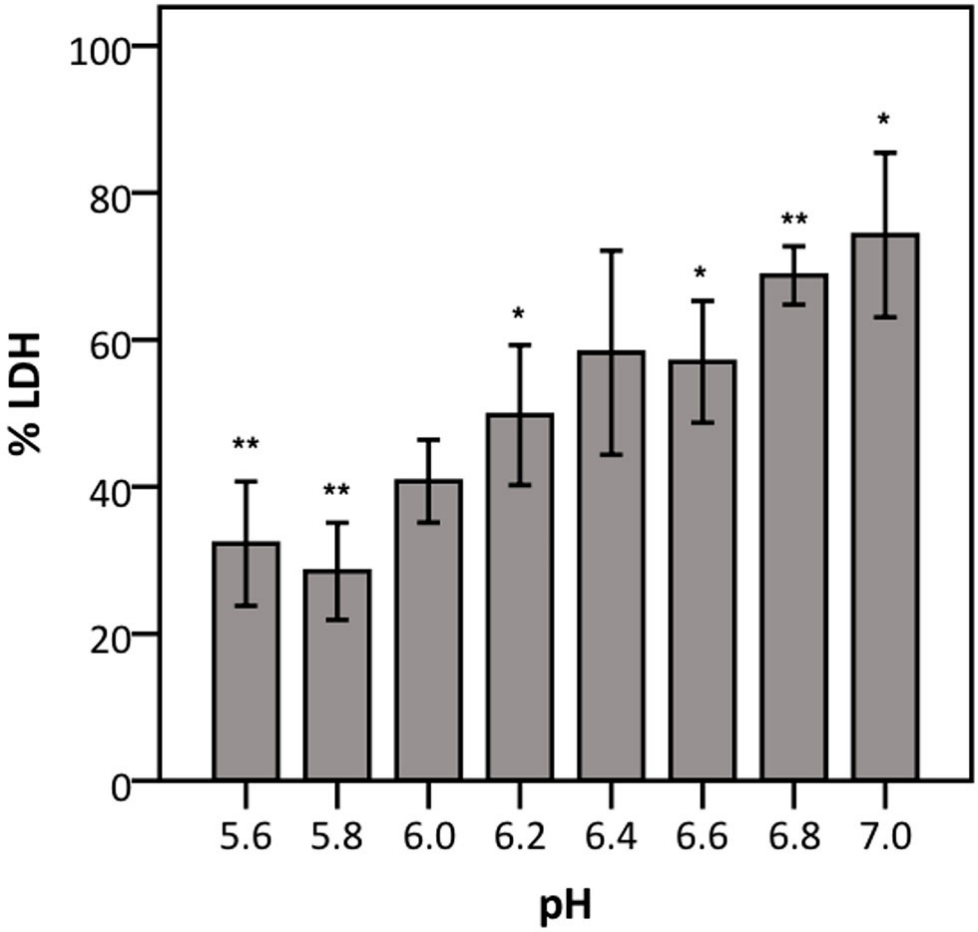
Influence of pH on LDH activity. The data are presented as a percentage compared to LDH activity in cell culture medium at pH 7.4–7.6. Error bars represent SE. Significant differences to 100% are indicated (*n* = 4, *p < 0.05, **p < 0.01; as determined by a one sample *t*-test or a Wilcoxon signed-rank test depending on the normality of the data).

**Figure 8:**
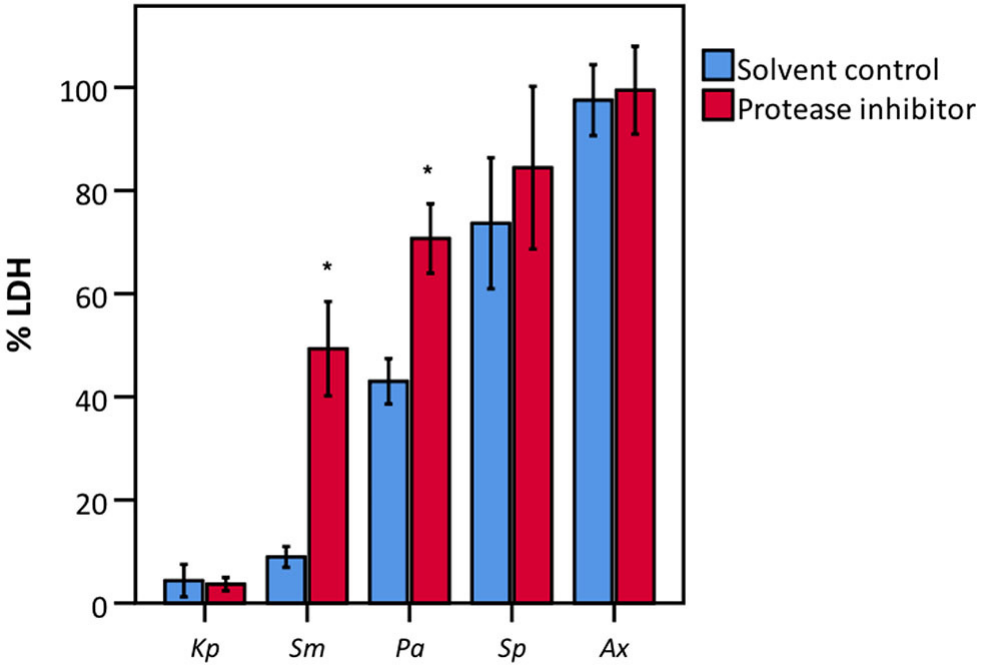
LDH activity after incubation with various bacteria in the presence of a bacterial protease inhibitor and of a suitable solvent control (DMSO). Data are presented as a percentage compared to a control without bacteria. Results are presented for *K. pneumoniae* (*Kp*), *S. maltophilia* (*Sm*), *P. aeruginosa* (*Pa*), *S. pneumoniae* (*Sp*), and *A. xylosoxidans* (*Ax*). Error bars represent SE. Significant differences between solvent control and protease inhibitor are indicated (*n* = 3–4, *p < 0.05; as determined by an independent sample *t*-test or a Mann-Whitney U-test depending on the normality of the data).

To elucidate whether there is a causal relationship between the pH decrease observed and decreased LDH activity, LDH activity was measured in cell culture medium with pH set between 5.6 and 7.0. After short exposure (30 min), LDH activity was determined. Results showed that at a pH of 6.2 and lower, LDH activity drops significantly to at least half of that at neutral pH (Figure 7). Combined with the results shown in Figures 4, 6, these findings support the hypothesis that low pH, due to *K. pneumoniae* and *S. pneumoniae* growth, affects LDH activity. However, this does not explain the influence of *P. aeruginosa* and *S. maltophilia* on LDH activity. For both species the main drop in LDH activity was observed after reaching stationary growth phase (Figures 4A,B), typically characterized by extensive protease production (Koch, 1985; Cezairliyan and Ausubel, 2017). To test the hypothesis that protease production by *P. aeruginosa* and *S. maltophilia* is responsible for the decline in LDH activity, a defined amount of LDH was exposed to the bacteria with the addition of a bacterial protease inhibitor cocktail. We also included *K. pneumoniae* and *S. pneumoniae* in this experiment to assess if bacterial proteases contributed to LDH interference, in addition to the above-described role of cell culture medium acidification. Again, *A. xylosoxidans* was added as a control since it did not affect LDH activity. In the presence of the protease inhibitor, LDH activity was less affected by *P. aeruginosa* and *S. maltophilia* as compared to the solvent control, and no effect of the protease inhibitor was observed for *K. pneumoniae, S. pneumonia*, and *A. xylosoxidans* (Figure 8). These results suggest that the influence on LDH activity by *P. aeruginosa* and *S. maltophilia* is, at least in part, mediated by protease production.

### A Modified Protocol for Cell Viability, Based on LDH Activity in the Viable Cell Fraction, Was Developed

**Figure 9:**
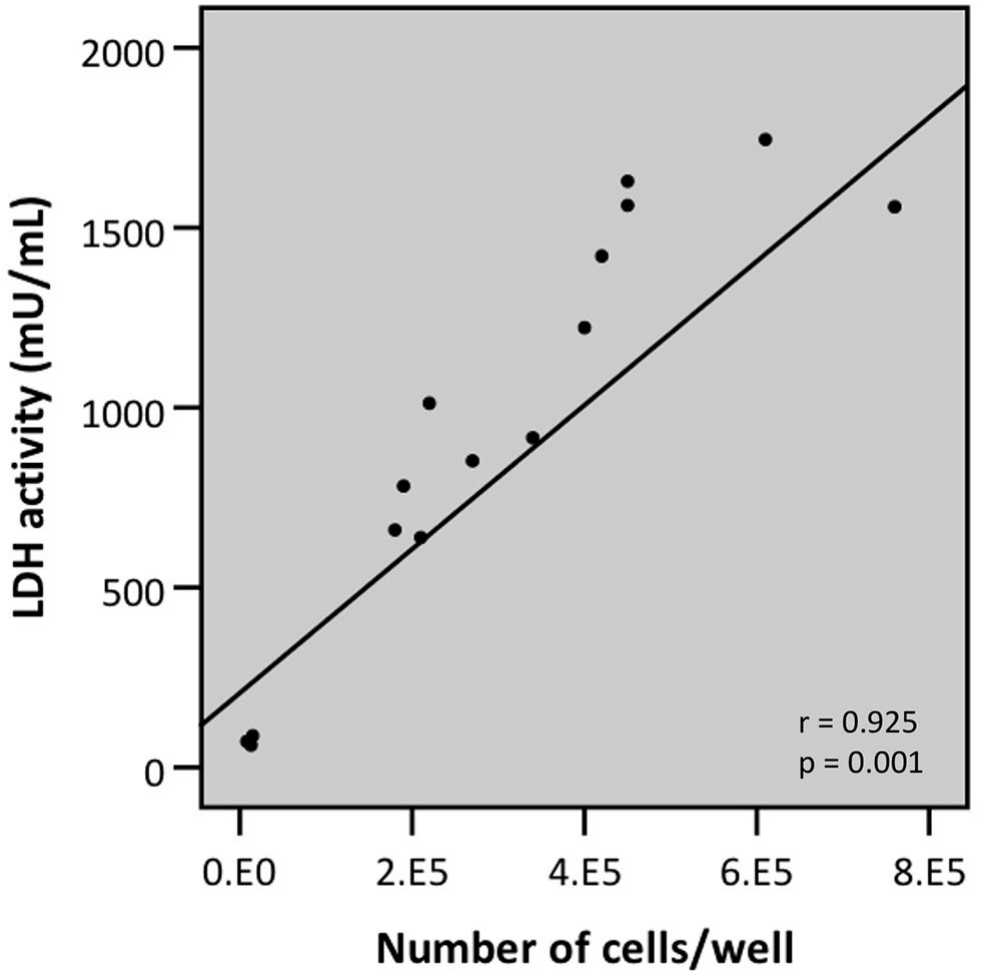
Linear correlation between the number of cells attached to the plastic surface (as determined by trypan blue staining followed by counting through light microscopy) and LDH activity as determined by LDH assay upon lysis of the airway epithelial cells (intracellular LDH assay) (*r*- and *p*-values were determined by a simple linear regression analysis).

To overcome possible bacterial interference, we subsequently developed a modified protocol for evaluating bacterial cell-mediated cytotoxicity using the LDH assay. This protocol, termed “intracellular LDH assay,” is based on the induced release of LDH from the viable cell fraction that is still attached to the plastic surface at the end of the infection experiment. Practically, after removing the bacteria-containing supernatant, the viable attached fraction is lysed by triton X-100-mediated cell lysis and only then LDH activity is determined. Two characteristics of this modified protocol were determined: the correlation between cell number, as determined by trypan blue staining, followed by light microscopy, and release of intracellular LDH; and the determination of the lower limit of detection (LLOD). Analysis by linear regression of cell number and intracellular LDH release revealed a strong linear correlation (*r* = 0.925) (Figure 9). This correlation can be described by the following linear equation:

$$\begin{array}{l} \text{LDH activity }\left(\text{mU}/\text{mL}\right)\;=\;0.002\;\times \text{ numberofcells }+\;207 \end{array}$$

**Figure 10:**
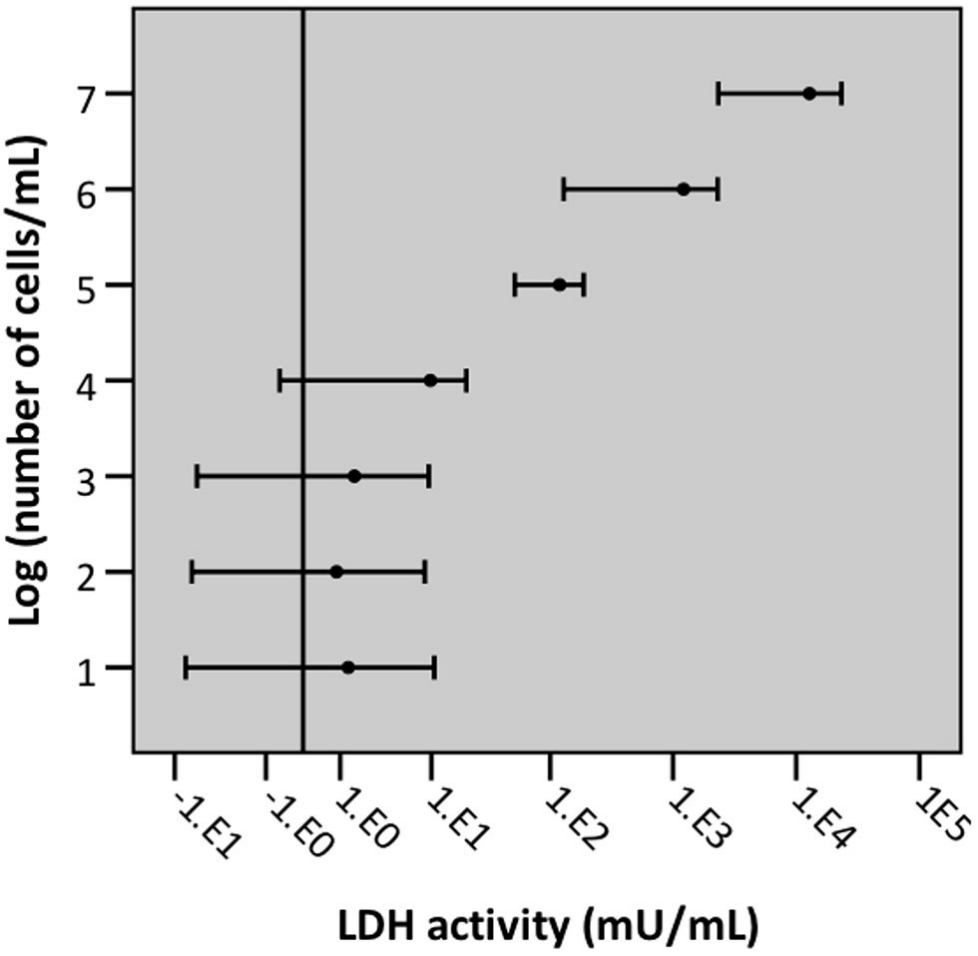
Graphical representation of the lower limit of detection of cells/mL with intracellular LDH assay. Error bars represent confidence intervals, with error bars not crossing the reference line (background) indicating that the signal was significantly different from background signal (*n* = 5, significance level 0.05).

To determine the LLOD (i.e., test value at which the signal can still be differentiated from background signal) a 10-fold dilution series of an A549 cell suspension (range: 1 × 10^7^ cells/mL −10 cells/mL) was lysed by exposure to triton X-100. Afterwards, LDH activity of the resulting supernatant was measured. The LLOD of the intracellular LDH assay is 1 × 10^4^ cells/mL (Figure 10).

### The Outcome of the Intracellular LDH Assay Correlates Strongly With Microscopic Imaging of Cell Detachment

**Figure 11:**
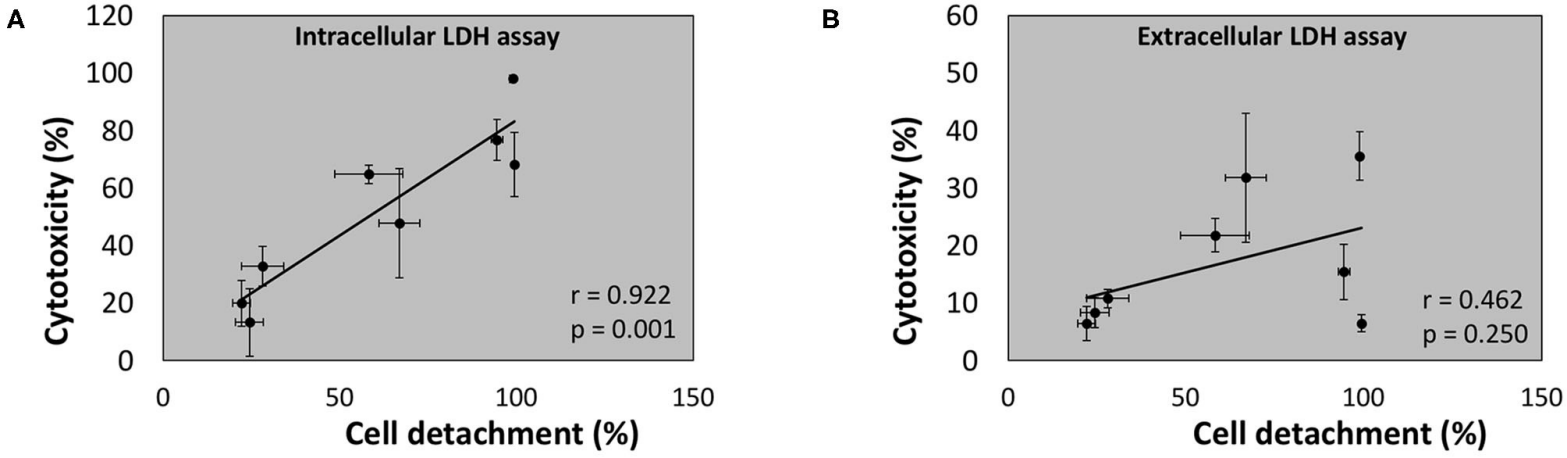
Linear correlation between cell detachment (%) as determined by epithelial cell nuclei enumeration and cytotoxicity (%) after 24 h of infection of A549 cells by eight bacterial species as determined by intracellular LDH assay **(A)** or extracellular LDH assay **(B)** (*n* = 5; *r*- and *p*-values were determined by a simple linear regression analysis on the averaged data per bacterial species).

Regression analysis of the correlation between the outcome of the conventional (“extracellular”) LDH assay and cell detachment indicates a poor (*r* = 0.462) and non-significant correlation (*p* = 0.250) (Figure 11B). However, a strong (*r* = 0.922) and significant (*p* = 0.001) correlation was found between the outcome of the newly developed intracellular LDH assay and cell detachment (Figure 11 A). These findings indicate that the intracellular LDH assay is able to predict cytotoxicity more accurately than the extracellular LDH assay.

## Discussion

The LDH cytotoxicity assay was first described in 1983 and has been extensively used since then in various fields (Korzeniewski and Callewaert, 1983). This assay is based on the spontaneous release of LDH, an intracellular enzyme, upon disruption of the cell membrane. Due to its straightforwardness and the possibility for high-throughput analysis, the LDH assay has been used in models to study lower airway respiratory tract infections with pathogens as *P. aeruginosa, S. pneumoniae, S. maltophilia, K. pneumoniae, M. catarrhalis*, and *S. aureus* (O'Grady et al., 2006; Slevogt et al., 2007; Victoria, 2009; Pompilio et al., 2010; Mairpady Shambat et al., 2015; Zahlten et al., 2015; Crabbé et al., 2017). In the present study, we evaluated the use of the LDH assay to quantify cytotoxicity induced by bacteria in airway epithelial cells after infection with eight bacteria encountered in the lower airways. Microscopic analysis of A549 monolayer integrity (reflected in the number of cells that remained attached to the culture flask) was consistent with previous studies reporting high cytotoxicity toward airway epithelial cells by *P. aeruginosa, S. aureus, K. pneumoniae*, and *S. maltophilia* (Hoskins et al., 2001; Hawdon et al., 2010; Karaba et al., 2013; Cheng et al., 2020) and low cytotoxicity for *A. xylosoxidans, S. pneumoniae*, and *M. catarrhalis* (Slevogt et al., 2007; Mantovani et al., 2012; Weight et al., 2019). Importantly, the results obtained indicate a discrepancy between cell detachment and cytotoxicity determined by the LDH assay for several bacterial species. Although, we cannot rule out a partial role of detached cells with an intact cell membrane in this discrepancy, we showed a strong interference of *P. aeruginosa, S. maltophilia, K. pneumoniae*, and *S. pneumoniae* with LDH activity, hereby contributing to their seemingly low cytotoxicity based on the conventional LDH assay. Indeed, for these bacterial species, low LDH activity in the supernatant during an infection does not necessarily correlate with a low cytotoxicity. Therefore, both cell detachment and cytotoxicity after infection with *P. aeruginosa, S. maltophilia, K. pneumoniae*, and *S. pneumoniae* were also determined in bronchial epithelial cells (BEAS-2B). Despite the clear interference with LDH activity by *K. pneumoniae* and *P. aeruginosa* as determined using the LDH enzyme, no discrepancy was observed between cell detachment and cytotoxicity in bronchial epithelial cells (BEAS-2B). For *K. pneumoniae* a possible explanation can be found in the low bacterial cell-mediated cytotoxicity toward bronchial epithelial cells. Second, LDH release by bronchial epithelial cells possibly occurs later in the cell death process whereas bacteria need a minimal contact time with the LDH enzyme before an effect on the activity can be observed (8 and 16 h for *K. pneumoniae* and *P. aeruginosa*, respectively).

The observed discrepancy between cell detachment and cytotoxicity after infection with bacteria that were found not to interfere with LDH activity (*A. xylosoxidans, R. mucilaginosa, S. aureus*, and *M. catarrhalis*) can possibly be explained by the fraction of detached cells with an intact cell membrane, a typical characteristic of early apoptotic cells (Caccamo et al., 2004). This fraction is considered as “viable” in the extracellular LDH assay and “dead” in the cell detachment data. Since a rinsing step was performed before fixation, loosely attached cells may be discarded. This leads to the apparent lower cell viability when focussing on cell attachment. The role of detached cells with an intact cell membrane in the experimental outcomes of epithelial cell infections is mostly not considered, and would need to be investigated in future studies. In addition, for *P. aeruginosa, S. maltophilia*, and *K. pneumoniae* it has been reported that the main process leading to cell death after 24 h of infection is necrosis rather than apoptosis (Yang et al., 2012; Roy et al., 2013; Nas et al., 2019). Thus, the difference between cell detachment and cytotoxicity for these bacteria is arguably a consequence of both bacterial interference with LDH activity and a high cytotoxicity toward lung epithelial cells, the latter being described previously (Cano et al., 2009; Karaba et al., 2013; Schaible et al., 2013). For *S. pneumoniae*, both apoptosis and necrosis occur (Schmeck et al., 2004). Here, the observed difference between cell detachment and cytotoxicity is thus most likely a consequence of detached cells with an intact cell membrane and bacterial interference with LDH activity.

The extent at which LDH levels are affected by bacterial species that interfered with LDH activity (*P. aeruginosa, S. maltophilia, K. pneumoniae*, and *S. pneumoniae*) depends on the bacterial density of the start inoculum, the duration of the infection and differs between strains of the same bacterial species. These data highlight the necessity to examine the applicability of the LDH assay for each specific experimental set-up.

We identified two possible mechanisms for bacterial interference with LDH activity. First, *K. pneumoniae* and *S. pneumoniae* affected LDH activity by lowering the pH of the culture medium below 6.2. We observed that at pH lower than 6.2, LDH activity decreases by half as compared to neutral pH. This low pH is not surprising as *S. pneumoniae* belongs to the lactic acid bacteria and *K. pneumoniae* is a known lactose fermenter (Seidler et al., 1975; Hoskins et al., 2001). Thus, both species are able to ferment lactose, and its precursors glucose and galactose, with acid end products as a result. Furthermore, in the cell culture medium used in this study (GTSF-2), galactose (2.52 mg/L) and glucose (3.52 mg/L) are present. Human LDH has an optimal stability and function at a pH of 7.2–7.4 (Gay et al., 1967). Although changes in pH are already known to influence LDH activity (Vallee and Williams, 1975), the LDH assay is still used to assess cytotoxicity in the presence of pH-altering bacteria as *K. pneumoniae* and *S. pneumoniae* (Cano et al., 2009; Zahlten et al., 2015; Lee et al., 2017; Brissac et al., 2018; Cheng et al., 2020) In contrast to *K. pneumoniae* and *S. pneumoniae, P. aeruginosa*, and *S. maltophilia* did not acidify the cell culture medium, indicating an alternative mechanism. Inhibiting proteases by a bacterial protease inhibitor cocktail resulted in higher LDH activity after exposure to *P. aeruginosa* and *S. maltophilia*, indicating that protease(s) are causing, at least in part, the observed interference with LDH activity. *P. aeruginosa* and *S. maltophilia* produce several proteases that could be responsible for the effect on LDH activity. However, proteolysis of LDH (bovine muscle and heart) by (chemo)trypsin and by cathepsin L, a serine and a cysteine protease, respectively, has been described before (Dabrowksa and Czapińska, 1990; Ohshita and Hiroi, 2006). Serine proteases produced by *P. aeruginosa* include protease IV, the large exoprotease (LepA), MucD and the *P. aeruginosa* small protease (PASP) (Okuda et al., 2011; Marquart and O'Callaghan, 2013; Milesi Galdino et al., 2017). In *S. maltophilia* three serine proteases have been described: StmPR1, StmPR2, and StmPR3 (Molloy et al., 2019). Although more in depth research is necessary, it can be hypothesized that one or more of these proteases could be responsible for the proteolysis of LDH. Furthermore, the observed differences in interference with LDH activity between strains of *P. aeruginosa* can possibly also be explained by strain-dependent differences in protease production (Milesi Galdino et al., 2017).

Given the problems associated with the use of the conventional (“extracellular”) LDH assay for select bacterial species, we developed a modified (“intracellular”) LDH assay, which can be applied regardless of bacterial interference with LDH activity. This protocol is based on the intracellular LDH of the attached, viable fraction of (airway) epithelial cells. By releasing intracellular LDH through induced cell lysis (using triton X-100), the number of attached and viable cells can accurately be determined. The intracellular LDH assay also has an acceptable lower limit of detection: cell numbers in infection experiments are rarely below 1 × 10 ^5^ cells/mL, which is 10 times above the LLOD (1 × 10 ^4^ cells/mL). Moreover, the results of the intracellular LDH assay correlate better with cell detachment data than the results of the extracellular LDH assay.

Importantly, our modified protocol also overcomes possible interference of bacterial LDH production, which has been reported for *R. mucilaginosa*. Specifically, LDH production by *R. mucilaginosa* was described in the presence of high levels of lactate, possibly explaining the absence of LDH production in our study as no high lactate levels are present (Lim et al., 2013).

To our knowledge, this is the first study reporting bacterial interference with LDH activity, leading to an underestimation of cytotoxicity in the presence of selected bacterial species. It needs to be emphasized that although this research focuses on lower airway bacteria and airway epithelial cells, the results concern all research fields that evaluate cytotoxicity in *in vitro* models to study host-pathogen interactions. Collectively, our data highlight the importance of verifying the reliability of the LDH assay in the specific experimental conditions and/or combining cytotoxicity assays with microscopic analysis. Furthermore, an altered protocol is proposed, which has been shown to be reliable in reflecting bacterial cell-mediated cytotoxicity in the presence of both bacteria interfering and not interfering with LDH activity.

## Data Availability Statement

The raw data supporting the conclusions of this article will be made available by the authors, without undue reservation.

## Author Contributions

AC and SV conceptualized the study and designed the experimental set-up. SV, EV, and LO performed the experiments. Data-analysis was performed by SV with contributions of AC and TC. SV, AC, and TC wrote the manuscript, with input from all authors. The manuscript was reviewed by all authors.

## Conflict of Interest

The authors declare that the research was conducted in the absence of any commercial or financial relationships that could be construed as a potential conflict of interest.
