# Supplementary figures and images for: Bacterial Interference With Lactate Dehydrogenase Assay Leads to an Underestimation of Cytotoxicity
Source: Front Cell Infect Microbiol. 2020 Sep 15;10:494. doi: 10.3389/fcimb.2020.00494 (PMC7523407; doi:10.3389/fcimb.2020.00494)

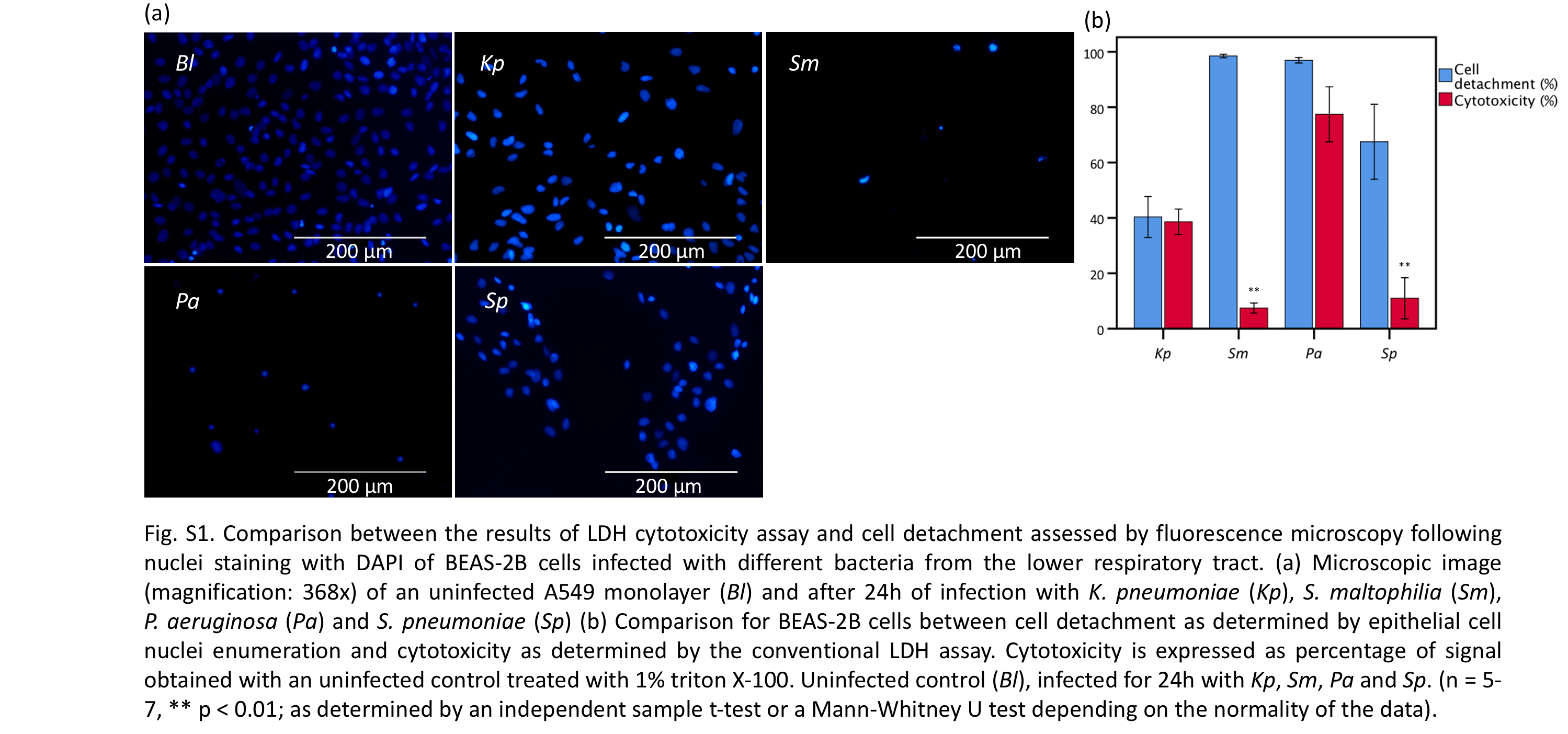

Supplement: Supplementary file 1 [file Image_1.JPEG]
